# Ultrasound-Guided Selective Nerve Root Block versus Fluoroscopy-Guided Interlaminar Epidural Block versus Fluoroscopy-Guided Transforaminal Epidural Block for the Treatment of Radicular Pain in the Lower Cervical Spine: A Retrospective Comparative Study

**DOI:** 10.1155/2020/9103421

**Published:** 2020-06-13

**Authors:** Jin Hyuk Jang, Woo Yong Lee, Jong woo Kim, Kyoung Rai Cho, Sang Hyun Nam, YongBum Park

**Affiliations:** ^1^Department of Rehabilitation Medicine, Sanggye Paik Hospital, Inje University College of Medicine, Seoul, Republic of Korea; ^2^Department of Anesthesiology, Sanggye Paik Hospital, Inje University College of Medicine, Seoul, Republic of Korea; ^3^Department of Family Medicine, Sanggye Paik Hospital, Inje University College of Medicine, Seoul, Republic of Korea; ^4^Department of Otorhinolaryngology Head and Neck Surgery, Sanggye Paik Hospital, Inje University College of Medicine, Seoul, Republic of Korea; ^5^Department of Plastic and Reconstructive Surgery, Sanggye Paik Hospital, Inje University College of Medicine, Seoul, Republic of Korea

## Abstract

**Background:**

Recently, ultrasound- (US-) guided selective nerve root block (SNRB) has been reported to have similar effects compared to fluoroscopy- (FL-) guided cervical epidural steroid injection (CESI). There is no published study comparing the therapeutic efficacy and safety of interlaminar- (IL-) CESI and transforaminal- (TF-) CESI with US-guided SNRB. Our retrospective study aimed to compare the mid-term effects and advantages of the US-guided SNRB, FL-guided IL-CESI, and TF-CESI for radicular pain in the lower cervical spine through assessment of pain relief and functional improvement.

**Methods:**

Patients with radicular pain in the lower cervical spine who received guided SNRB (*n* = 44) or FL-guided IL (*n* = 41) or TF-CESI (*n* = 37) were included in this retrospective study. All procedures were performed using a FL or US. The complication frequencies during the procedures, adverse event, treatment effects, and functional improvement were compared at 1, 3, and 6 months after the last injection.

**Results:**

Both the Neck Disability Index (NDI) and Verbal Numeric Scale (VNS) scores showed improvements at 1, 3, and 6 months after the last injection in all groups, with no significant differences between groups (*P* < 0.05). Furthermore, the treatment success rate at all time points was not significantly different between groups. Logistic regression analysis revealed that the injection method (US- or FL-guided), cause, sex, age, number of injections, and pain duration were not independent predictors of treatment success. Blood was aspirated before injection in 7% (*n* = 3), 14% (*n* = 6), and 0% patients in the FL-guided IL, TF, and US-guided groups, respectively. In 2 patients of FL-guided IL and 7 of FL-guided TF group, intravascular contrast spread was noted during injection.

**Conclusions:**

Our results suggest that, compared with FL-guided IL and TF-CESI, US-guided SNRB has a low intravascular injection rate; it is unlikely that serious complications will occur. Also, US-guided SNRB requires a shorter administration duration while providing similar pain relief and functional improvements. Therefore, for the treatment of patients with lower cervical radicular pain, US-guided SNRB should be considered as a prior epidural steroid injection.

## 1. Background

Cervical radiculopathy is a common condition with an annual incidence of 83 per 100,000 [1]. When initial conservative treatment fails, the physician can consider an epidural steroid injection (ESI), which is administered in the cervical spine via an interlaminar (IL) or a transforaminal (TF) route [2, 3]. In general, cervical epidural steroid injections (CESIs) have been associated with significant complications and are a subject of intense debate [3].

Recently, various study groups, including ours, have shown the reliability of ultrasound (US) guidance for cervical selective nerve root block (SNRB) [4–7]. Because the main advantage of US is direct real-time visualization of soft tissue structures, bony surfaces, and needle manipulation, it is particularly beneficial for injections into the cervical spine, where a multitude of vulnerable vessels and other vital soft tissue structures are compacted in a small area and often in the path of the projected needle trajectory [8]. In addition, this broadly available method does not expose patients to ionizing radiation or result in contrast medium-related allergic reactions [7].

Since Galiano et al. [4] reported the use of US-guided cervical SNRB in 2005, several studies have reported the feasibility and effectiveness of this procedure [4–6]. However, these studies did not compare the US-guided technique with other methods such as fluoroscopy- (FL-) guided TF-ESI or IL-ESI. Therefore, it was difficult to draw conclusions regarding the usefulness of US-guided injection methods. Jee et al. [7] compared short-term treatment effects between US-guided cervical SNRB and FL-guided TF-ESI and found no significant intergroup differences. In addition, Park et al. [9] found that pain relief and functional improvements were similar with FL-guided IL-ESI and US-guided SNRB.

To the best of our knowledge, no published study has compared the therapeutic efficacy and safety of FL-guided IL-CESI and TF-CESI with those of US-guided SNRB. Accordingly, the aim of this retrospective study was to evaluate and compare the mid-term (6 months) effects of US-guided cervical SNRB and FL-guided TF-CESI/IL-CESI on pain and function in patients with lower cervical radicular pain. In addition, safety and procedure times were assessed as secondary outcomes.

## 2. Methods

### 2.1. Ethics

This was a retrospective comparative review of chart data. Patient privacy and data confidentiality were maintained throughout the research process. The institutional review board of the corresponding author's affiliated university approved the study. The approval included a waiver of informed consent because there was no direct contact with the study population and all patient identifiers were removed from the dataset on initial collection.

### 2.2. Subjects

Potential study participants were patients who received US-guided SNRB or FL-guided TF-ESI/IL-ESI at our outpatient rehabilitation department and pain clinics between 2015 and 2017. On the day of the procedure, before injection, all patients were requested to fill out self-assessment questionnaires regarding their baseline information (e.g., pain level and functional status). The electronic clinical records and questionnaire responses were retrospectively reviewed for the collection of data and determination of compliance with the inclusion criteria for the study.

We selected patients aged >18 years who received US-guided SNRB or FL-guided TF-ESI/IL-ESI for the treatment of lower cervical radicular pain that lasted for at least 3 months. Cervical radicular pain was diagnosed on the basis of clinical profiles, medical examinations, and the confirmation of disc herniation or spinal stenosis via cervical computed tomography or magnetic resonance imaging. None of the patients had responded to conservative treatments administered for at least 4 weeks, including analgesic use (nonsteroidal anti-inflammatory drugs (NSAIDs) or opioids) and physical therapy. The score for self-reported neck pain on the Verbal Numeric Scale (VNS) was at least 5 points, with symptoms present on most days for at least 3 months. The exclusion criteria included psychiatric disorders, neurological deficits, cervical myelopathy, laboratory results suggestive of inflammatory disease or rheumatoid disorders, and a history of cervical spine surgery.

### 2.3. Injection Methods

US-guided SNRB or FL-guided TF-ESI/IL-ESI is commonly used for the treatment of symptomatic lower cervical radicular pain at our institution. The patient is provided detailed information about the procedure and the expected benefits and risks, following which consent is obtained.

**Figure 1:**
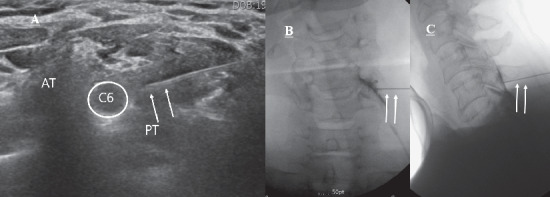
(A) Ultrasonography-guided selective nerve root block (target nerve root: C6). The needle (arrow) is placed on the dorsal surface of the C6 nerve root. AT, anterior tubercle; PT, posterior tubercle. (B) C6 transforaminal epidural injection. The A-P view of the contrast media which spread into the intraforaminal lesion. (C) C5-6 interlaminar epidural injection. Lateral view of the contrast media which spreads into the epidural space. The arrow indicates a needle.

The procedures were performed by two physicians (YP and YL, who have more than 8 years of experience in US- and FL-guided procedures). All treatments were performed as outpatient procedures. Accuvix XQ® (Samsung Medison, Seoul, Korea) with a 612 MHz linear probe was used as the US instrument [4–6]. The patients were instructed to rotate their heads 30°40° away from the targeted area in the supine position [6], and the frontal cervical spine area from the clavicle to the mandible was adequately disinfected with betadine and covered with an aseptic dressing. The C5C7 nerve roots were identified on US by the shape of the transverse process; the C5 and C6 transverse processes both have obvious anterior and posterior tubercles, while the C7 transverse process has a rudimentary anterior tubercle and a prominent posterior tubercle [5–8]. The targeted transverse process was identified by slow movement of the probe in all directions, with the C7 transverse process as a reference point [7]. The optimal image of the nerve root, location of the radicular artery, and surrounding vessels near the border of the nerve root were obtained through probe manipulation in the power and color Doppler modes. Next, via an in-plane approach with the free-hand technique, a 50 mm 23 G needle was inserted toward the nerve root in the posterior to anterior direction on the short axis view. The needle end was placed on the dorsal side of the nerve, with care to avoid damage to the deep cervical artery near the insertion site (if present) and location of the area free of the radicular artery [7]. Then, 1 ml of 1% lidocaine was injected, following which the patient was monitored for the onset of clinical manifestations such as mid-neck and contralateral arm pain, metallic taste, dizziness, tachycardia, full-body paresthesia, auditory changes, slurred speech, and motor ataxia for 1 to 2 minutes [10]. On confirmation of the absence of abnormal findings and careful aspiration, 3 cc of the treatment drug, which comprised 2 ml of dexamethasone (10 mg) and 1 ml of 0.5% lidocaine, was injected under real-time US guidance (Figure 1(a)).

The FL-guided TF-ESI procedure was performed by the same physician. All treatments were performed on an outpatient basis. The patient positioning and disinfection procedure for the injection site were identical to those described for US-guided SNRB.

The fluoroscopic apparatus KMC 950 (KOMED, Kwangju, Korea) was adjusted such that a proper oblique and well-defined view of the intervertebral foramen was obtained. Following the application of 1% lidocaine for local anesthesia at the targeted area, the distal 5 mm end of a 22 G Spinocan® needle (BRAUN, Melsungen, Germany) was bent by approximately 15°20°. The angulated needle end was carefully tilted toward the posterior side until it contacted the medial surface of the superior articular process, which formed the posterior side of the targeted foramen. When the needle end contacted the superior articular process, the needle was rotated by 180° and advanced forward for 2-3 mm under continuous FL guidance. The depth of the fluoroscopic apparatus was adjusted under the anteroposterior view for location of the needle end at the center of the articular pillar. Cerebrospinal fluid and blood absorption tests were performed for the detection of possible blood traces in nontargeted areas. For confirmation of shadowed contrast of the foramen, nerve root, epidural space, and other related structures, fluoroscopic images were continuously monitored during injection of the nonionic contrast medium Omnipaque 300 (GE Healthcare, Carrigtohill, Ireland). Then, 1 ml of 1% lidocaine was injected once the needle was repositioned for vessel infusion, the images of the nerve root showed appropriate shadowed contrast, and the contrast medium was not identified within any proximal vessel. The patients were monitored for the onset of clinical manifestations, such as mid-neck and contralateral arm pain, metallic taste, dizziness, tachycardia, full-body paresthesia, auditory changes, slurred speech, and motor ataxia for 1 to 2 minutes after the injection [10]. On confirmation of the absence of abnormal findings, the treatment drug was injected, and the procedure was completed (Figure 1(b)).

For FL-guided IL-CESI, the patient was placed in the prone position. A pillow was placed under the chest for shoulder elevation, which helps in widening the IL space by flexing the spine. The KMC 950 (KOMED) was used for all injections. The skin of the posterior neck was prepared and draped in sterile fashion, and the epidural space between C5 to C7 and T1 was entered. A 22 G Spinocan® needle (BRAUN) was then advanced into the posterior epidural space via a midline approach. If the needle was just posterior to the spinolaminar line on the lateral view, it was advanced very carefully by twirling and opening of the ligamentum flavum with intermittent injection of Omnipaque 300 (GE Healthcare) until it spread smoothly in the epidural space. When the contrast material was spreading in the epidural space, needle advancement was stopped and the location of the needle tip in the epidural space was confirmed by an additional test injection of contrast agent. Then, 3 cc of the treatment drug, which comprised 2 ml of dexamethasone (10 mg) and 1 ml of 0.5% lidocaine, was injected into the epidural space (Figure 1(c)).

The patients were scheduled to receive two consecutive therapeutic injections, with a 2-week interval between injections. If the initial injection resulted in significant symptom reduction (VNS ≥ 50%), the second injection was omitted. If there was no pain relief or the pain worsened, or if the patient satisfaction rating was equal to or below “fair,” the second injection was not considered. If the patient satisfaction score was “good” despite a VNS score improvement of <50%, the second injection was scheduled.

Because none of the patients had shown any improvement with medications such as analgesics (NSAID or opioid) and physical therapy for 4 weeks, we did not set any limit for continuation of previous exercise programs and drug therapy or return to work. No specific physical therapy, occupational therapy, bracing, or other specific intervention was utilized.

### 2.4. Review of Clinical Data

A standardized chart abstraction form was used for the extraction of data regarding demographics, treatments, pain severity, analgesic use, and functional assessments. A nursing personnel not involved in the procedure conducted follow-up interviews during a hospital visit at 1, 3, and 6 months after the last injection.

Primary outcomes included Neck Disability Index (NDI) scores and VNS scores for pain, which were recorded before injection and at 1, 3, and 6 months after the last injection. The degree of physical disability was measured using NDI, which is the most widely used questionnaire survey for the assessment of cervical spine abnormalities. NDI was first developed for evaluation of the degree of limitations in the daily lives of patients with severe cervical pains, particularly those with whiplash trauma [11]. It includes 10 questions related to functional activity (seven questions), symptoms (two questions), and concentration (one question) [11]. The final score is obtained by adding the scores for all questions. A higher NDI score indicates increased functional disability related to cervical abnormality. The original developer, Vernon, suggested score interpretation as follows: ≤4 or lower, no disability; 514, mild disability; 1524, moderate disability; 2534, severe disability; and ≥35, complete disability [11]. For calculation of VNS scores, the patients were asked to rate their pain on a scale from 0 to 10, where 0 and 10 represented no pain and the worst pain possible, respectively. Scores were assigned as whole numbers [12].

The patient satisfaction score was calculated using a five-point scale at 2 weeks after the first injection: <0, no effect at all; 1, bad; 2, fair, 3, good; and ≥4, excellent. The meaning of each satisfaction level was as follows: excellent, “satisfied with the treatment result as expected”; good, “not as much as expected but willing to try this treatment next time when pain redevelops”; fair, “had some effect but not enough to choose the same treatment next time when pain redevelops”; and bad, “same effect as that of the prior treatment or worse” [7].

Patients with a VNS score improvement of >50% and NDI score improvement of >40% were categorized as responders [11, 12]. Patients who did not show this degree of improvement and those who required reinjection, invasive procedures, or surgery during the follow-up period after injection were categorized as nonresponders. Their VNS and NDI scores were recorded for statistical analysis and subsequently excluded. Independent variables, including the injection method, the number of injections, analgesic use, sex, the pain duration, and age, were documented in the medical charts. Age was categorized into five groups as follows: <39, 40–49, 50–59, 60–69, and >70 years.

The procedure duration was also recorded. For US-guided SNRB, the procedure duration was defined as the time interval between contact of the US probe with the patient's skin and completion of injection [13, 14]. For FL-guided IL-ESI/TF-ESI, the procedure duration was defined as the temporal interval between acquisition of the first radiographic image and completion of the second injection [13, 14].

Finally, we reviewed the charts for immediate adverse events such as vasovagal reaction, facial flushing, and brief severe neck pain within a few minutes after injection. The patients were handed a questionnaire at the end of the procedure, which had to be completed within 48 h and returned at the 2-week follow-up visit.

### 2.5. Statistics

Pearson's chi-square test and one-way analysis of variance (ANOVA) were used to compare variables such as sex, age, body mass index (BMI), number of injections, analgesic use, anticoagulant use, and the pain duration among the three groups. At each time point (before injection and 1, 3, and 6 months after the last injection), VNS and NDI scores were compared using repeated measures ANOVA with Bonferroni's correction for post hoc comparisons. Pearson's chi-square test was used to test differences in proportions, while one-way ANOVA was used to compare the procedure duration among groups. Univariate and multivariate logistic regression analyses with Pearson's chi-square test were used to determine whether the injection method, age, sex, analgesic use, and the number of injections were independent predictors of treatment success. All statistical analyses were performed using SAS Enterprise Guide 4.1 (4.1.0.471). A *P* value of <0.05 was considered statistically significant.

## 3. Results

**Figure 2:**
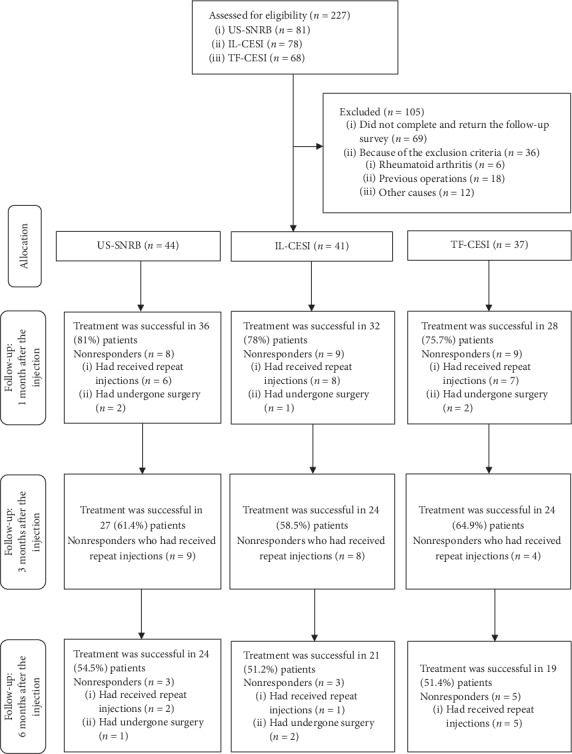
Flow diagram indicating progress of patients through the study. US-SNRB: ultrasonography-guided selective nerve root block, IL-CESI: fluoroscopy-guided interlaminar cervical epidural steroid injection, TF-CESI: fluoroscopy-guided transforaminal cervical epidural steroid injection.

A total of 227 patients, including 81, 78, and 68 who received US-guided SNRB, FL-guided IL-CESI, and FL-guided TF-CESI, respectively, had received injections during the study period. From these, 69 (30.3%) patients were excluded because they did not complete and return the follow-up surveys. Another 36 (15.8%) were excluded on grounds of the exclusion criteria. Eighteen patients (7.9%) who had previously undergone surgeries and six (2.6%) with underlying rheumatoid arthritis were also excluded. 12 (5.2%) patients were excluded as other causes. Eventually, 122 (53.7%) patients, including 44, 41, and 37 who received US-guided SNRB, FL-guided IL-CESI, and FL-guided TF-CESI, respectively, were included (Figure 2).

The average age of patients in the US-guided SNRB, FL-guided IL-CESI, and FL-guided TF-CESI was 52.9 ± 11.9, 54.8 ± 10.3, and 56.0 ± 9.8 years, respectively, with no significant differences among the three groups. Moreover, there were no significant intergroup differences in sex, BMI, the number of injections, the etiology (herniated cervical disc or stenosis), target nerve root, analgesic use, anticoagulant use, and the pain duration (Table 1).

**Figure 3:**
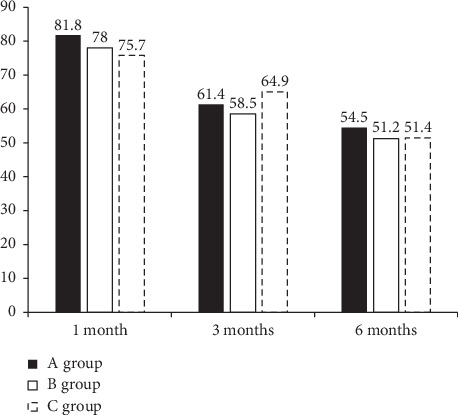
Illustration of significant pain relief (VNS score improvement of >50%, NDI score improvement of >40%). *A* group: ultrasound-guided selective nerve root block, *B* group: fluoroscopy-guided interlaminar epidural block, *C* group: fluoroscopy-guided transforaminal epidural block. Verbal Numeric pain Scale (VNS), Neck Disability Index (NDI).

NDI and VNS scores showed a significant improvement at 1, 3, and 6 months after the last injection in all groups, with no significant differences at any time point (Table 2). The proportions of patients with a VNS score improvement of >50% and an NDI score improvement of >40% are shown in Figure 3; there were no significant intergroup differences at any time point. At 1 month, six patients had received repeat injections and two had undergone surgery in the US-guided SNRB group. Thus, the 1-month treatment success rate was 81.8% (*n* = 36) in this group. In the FL-guided IL-CESI and FL-guided TF-CESI groups, eight and seven patients, respectively, had received repeat injections, and one and two patients, respectively, had undergone surgery at 1 month. Thus, the 1-month treatment success rates were 78% (*n* = 32) and 75.7% (*n* = 28), respectively. At 3 months, nine, eight, and four patients had received repeat injections in the US-guided SNRB, FL-guided IL-CESI, and FL-guided TF-CESI groups, respectively, with corresponding treatment success rates of 61.4% (*n* = 27), 58.5 (*n* = 24), and 64.9% (*n* = 24). At 6 months, two patients had received repeat injections and one had undergone surgery in the US-guided SNRB group, one patient had received repeat injections and two had undergone surgery in the FL-guided IL-CESI group, and five patients had received repeat injections in the FL-guided TF-CESI group (Figure 2). Thus, the final 6-month treatment success rates were 54.5% (*n* = 24), 51.2% (*n* = 21), and 51.4% (*n* = 19), respectively (Figure 3). There were no significant differences in the success rate at any time point among the three groups. Moreover, there was no clinically significant decrease in the proportion of analgesic (NSAIDs and opioid) users at 6 months in all three groups.

The proportions of patients with >50% improvement in the VNS score and >40% improvement in the NDI score are illustrated in Figure 3. The respective rates at 6 months were 54.5%, 51.2%, and 51.4% in the US-guided, FL-guided IL, and FL-guided TF groups. There were no significant differences at any time point between the three groups.

Univariate and multivariate logistic regression analyses revealed that the injection method, the etiology (herniated cervical disc, stenosis), sex, age, the number of injections, and the pain duration were not independent predictors of treatment success (*P* > 0.05; Tables 3 and 4).

In US-guided SNRB, the mean procedure time was 223 seconds, which was shorter than that of FL-guided IL-CESI (383 seconds) or TF-CESI (382 seconds) group.

Immediately after the procedure, five patients in the US-guided group, three in the FL-guided IL-CESI group, and four in the FL-guided TF-CESI group experienced vasovagal reactions, while three, three, and four patients, respectively, developed a transient headache. Overall, at the 2-week follow-up session, two patients in the US-guided group, two in the FL-guided IL-CESI group, and three in the FL-guided TF-CESI group reported transient pain exacerbation (in the head or upper limbs) at 48 h after the procedure. No patient reported headache suggestive of postpuncture syndrome, and no cases of infection or hematoma were recorded during the 2-week follow-up period. Blood was aspirated before injection in 14.6% (*n* = 6), 13.5% (*n* = 5), and 0% patients in the FL-guided IL-CESI, FL-guided TF-CESI, and US-guided SNRB groups, respectively. Intravascular contrast spread was noted during injection in seven patients in the FL-guided IL-CESI group and eight in the FL-guided TF-CESI group.

## 4. Discussion

In this retrospective study, US-guided SNRB, FL-guided TF-CESI, and FL-guided IL-CESI resulted in clinically meaningful and significant improvements in all parameters at the end of a mid-term period of 6 months after the last injection, with treatment success rates of 54.5%, 51.2%, and 51.4% in the US-guided SNRB, FL-guided TF-CESI, and FL-guided IL-CESI groups, respectively. The treatment success rates at 1, 3, and 6 months after the last injection were comparable among groups. However, the duration of US-guided SNRB was shorter than that of the FL-guided procedures. We speculate that the greater efficiency conferred by US guidance stems from the inherent difficulties related to FL. Pain control and functional improvements were similar in all three groups. To the best of our knowledge, this is the first study to compare the treatment efficacy and the injection efficiency with regard to the procedure duration among US-guided SNRB, FL-guided IL-CESI, and FL-guided TF-CESI over a 6-month follow-up period in patients with lower cervical radicular pain.

ESI is currently used for the treatment of cervical radicular pain [15]. Two approaches are used to access the epidural space, namely, the IL and TF approaches. Although FL-guided IL-ESI and TF-ESI are both common techniques used for the management of radicular pain, the superior of the two in terms of efficacy remains unknown [16]. The advantage of FL-guided TF-ESI over FL-guided IL-ESI is enhanced delivery of medication to pain generators in the ventral epidural space [16]. Therefore, TF injection requires a smaller dose of epidural steroids for pain management [16, 17]. However, FL-guided TF-CESI is associated with certain unique risk factors and often causes permanent, severe complications, including spinal cord infarction, paralysis, disc weakening, and discitis [16, 18, 19]. Moreover, the TF technique does not decrease the risk of known complications attributed to IL, including hematoma formation, subdural or dural punctures, and cervical myelopathy [20]. According to existing data, the long-term efficacy of TF-ESI is greater than that of IL-ESI. Nonetheless, data regarding benefits common to both methods are conflicting. A recent systematic review revealed that the efficacy of both techniques in terms of functional improvements and pain relief is not significantly different despite the risks associated with each method [21]. Several previous studies of US-guided SNRB reported only temporary minor complications and an efficacy similar to that of both FL-guided TF-ESI and FL-guided IL-ESI, with no major complications such as spinal cord or brain infarction, paralysis, infection, and death [7, 9, 22]. However, these results are limited because of the small number of studies, and further studies need to determine the safety of CESI procedures.

As mentioned earlier, FL-guided TF-ESI has been associated with rare but devastating complications, including paralysis and death [23]. The injection of particulate steroid into the anterior spinal artery can lead to catastrophic infarction of the spinal cord and anterior spinal artery syndrome, which is characterized by complete motor paralysis with the loss of pain and temperature senses and preservation of position, vibration, and motion senses in the posterior columns [24]. FL-guided IL-ESI also has the potential for intravascular injection, which could lead to more serious adverse effects [25]. A single case report of quadriparesis after injection at the C6-7 level has been reported, and a vascular event was postulated as the cause [26]. In the present study, symptoms of intravascular injection were not identified during US-guided SNRB in any case, whereas five and six patients in the FL-guided TF-CESI and FL-guided IL-CESI groups, respectively, exhibited such symptoms. In previous studies, intravascular injection rates of 26%32.8% and 4.2%9% have been reported for TF-ESI and IL-ESI, respectively [27–30]. The main advantage of the US approach is real-time visualization of vulnerable vessels and other vital soft tissues in and around the projected needle trajectory [7]. In the present study, although there were no intravascular events associated with US-guided SNRB, consistent with findings in previous studies, there was no statistically significant difference in the rate of intravascular injection among the three groups [7, 9, 22]. Therefore, large-scale, multicenter, prospective, comparative studies are necessary to clarify our findings.

Although US guidance can help in avoiding intravascular injections, it may not be able to detect such injections [11]. To prevent complications associated with intravascular injections, two approaches should be used. First, a nonparticulate steroid should be injected. Second, an anesthetic test dose must be injected first as a precautionary measure [7, 9, 22]. Previous studies successfully avoided intravascular injection by implementing both these measures during US-guided SNRB [7, 9, 22].

In the present study, the treatment effect was similar for all three methods. It is hypothesized that the injected drug may not enter the lesion sufficiently during US-guided SNRB because the target point is more distal than that for FL-guided injection; this can reduce the effectiveness of the treatment. Jee et al. [7] reported that only approximately 30% injections resulted in the typical epidural spread that can be achieved under fluoroscopic guidance. In another study, 1 ml of contrast agent was observed in the proximal spinal canal and the intraspinal epidural space in 29.7% patients who received US-guided SNRB [22]. However, despite differences in the injection target and the spread pattern of the contrast medium, studies comparing the treatment efficacy between FL-guided and US-guided injections found no significant differences [7, 9, 22]. These results can be explained by several hypotheses. Yamauchi et al. [6] have attributed these results to hydrostatic pressure and osmotic effects, which lead to further absorption of the treatment solution into the nerve fibers. Second, differences in the viscosity of the injected drug and contrast medium could have led to these results [7]. Jee et al. [7] observed spread of the contrast medium into the intraforaminal epidural space by comparing anteroposterior images obtained after contrast medium injection and washout images obtained after treatment drug injection in 25 patients. Despite its relatively high viscosity, [31] the contrast medium (Omnipaque) was observed in the infraspinal epidural area after washout. This could be explained by the low viscosity of the injected drug (1 ml of 0.5% lidocaine + 1 mg of dexamethasone), which may have diluted and further spread the contrast medium into the targeted area [7]. Such phenomena may facilitate proximal delivery of the drug to the lesion for greater efficacy [7, 22].

Another main advantage of US-guided SNRB, in addition to no radiation exposure, is direct real-time visualization of vessels and nerves, which is particularly beneficial for injection in areas packed with a multitude of vulnerable vessels and other vital soft tissue structures that are compacted in a small area and are often in the path of the projected needle trajectory [8, 32]. In addition to the advantage offered by the US-guided SNRB, there is no difference in the treatment effect for 6 months, compared to the FL-guided method. Furthermore, the use of intravascular injection can be avoided, and the procedure time is shorter than that of the FL-guided method. In view of all these advantages, it is considered that the US-guided SNRB may be the first choice for clinicians when considering lower cervical steroid injection as a treatment method.

However, US-guided SNRB also has several disadvantages compared to the FL-guided methods. First, Although US may assist in avoiding intravascular injections, it is not clear if it can help to detect such injections [7–9]. To solve these limitations, color doppler US and lidocaine tests can be performed during the US-guided SNRB, but it is thought that the detection ability of intravascular injection will be inferior to the injection method using digital subtraction angiography and live video fluoroscopy. In addition, the dose used in the lidocaine test in this study is small volume, so there may be no symptoms of intravascular injection. As a result, undetected critical vessel with US does not necessarily ensure the absence of the vessel [7]. Second, the technique and the image are quite operator-dependent [33]. The practitioner requires experience to obtain a good image and direct the needle safely to the target structure [33]. Third, using US alone, practitioners cannot confirm the level that the injectate has reached (dorsal root ganglion or epidural space) [7]. Therefore, it may be necessary to study how to compensate for these shortcomings in the future.

This study has several limitations. First was the retrospective design. Although patients were selected on the basis of extensive inclusion and exclusion criteria, there may have been heterogeneity in the selected sample. Nevertheless, patient demographics and the clinical and imaging parameters before treatment and at each follow-up visit are documented in a standardized format in the case records of patients receiving injections at our institution. Second, we could not entirely prevent the use of other treatments such as medication and physical therapy during the follow-up period. Third, we did not use FL to confirm appropriate injection of the drug into the targeted area during US-guided SNRB, and this could have affected the results. Further studies that address these limitations are necessary.

## 5. Conclusion

In summary, the findings of this study suggest that the outcomes of US-guided SNRB and FL-guided IL-CESI/TF-CESI for lower cervical radiculopathy are similar in terms of pain reduction and functional improvements. However, US-guided SNRB is not associated with radiation exposure and requires lesser time. Thus, it helps clinicians in identifying and avoiding vessels in and around the injection trajectory. Accordingly, US-guided SNRB could be considered as the first choice of treatment for lower cervical radicular pain. However, although intravascular events can be avoided by using the US-guided approach, confirmation of the absence of small critical vessels may not be possible with the current US technology. Further studies are necessary to clarify our findings.

## Figures and Tables

**Table 1:** General characteristics of the patients.

	Ultrasound-guided SNRB (*n* = 44)	Fluoroscopy-guided IL-ESI (*n* = 41)	Fluoroscopy-guided TF-ESI (*n* = 37)
Age (years)	52.9 ± 11.9	54.8 ± 10.3	56.0 ± 9.8
Sex, *n* (%)			
Female	32 (72.7)	27 (65.9)	24 (65.9)
Male	12 (27.3)	14 (34.1)	13 (35.1)
BMI (kg/m^2^)	24.09 ± 2.33	24.36 ± 2.99	23.83 ± 2.60
Number of injections	1.43 ± 0.50	1.46 ± 0.50	1.41 ± 0.49
Cause, *n* (%)			
HCD	14 (31.8)	16 (39.0)	15 (40.5)
Stenosis	30 (68.2)	25 (61.0)	22 (59.5)
Target nerve root, *n* (%)			
C5	11 (25.0)	8 (19.5)	7 (18.9)
C6	22 (50.0)	23 (56.1)	21 (56.8)
C7	11 (25.0)	10 (24.4)	9 (24.3)
Analgesic use, *n* (%)			
NSAID usage	29 (65.9)	21 (51.2)	20 (54.1)
Opioid usage	27 (61.4)	21 (51.2)	20 (54.1)
Anticoagulant use, *n* (%)	8 (18.2)	6 (14.6)	6 (16.2)
Pain duration (month)	6.80 ± 2.16	6.61 ± 2.21	6.68 ± 2.05

Values are mean ± standard deviation. Selective nerve root block (SNRB), transforaminal (TF), interlaminar (IL), epidural steroid injection (ESI), body mass index (BMI), herniated cervical disc (HCD), non-steroidal anti-inflammatory drugs (NSAIDs).

**Table 2:** Comparison of VNS and NDI from baseline to 1, 3, and 6 months after last injection.

		Baseline	1 month	3 months	6 months
VNS	US-SNRB	6.33 ± 1.06	2.41 ± 1.03^*∗*^	2.98 ± 1.97^*∗*^	2.57 ± 1.10^*∗*^
	FL-CIESI	6.22 ± 0.86	2.64 ± 1.33^*∗*^	2.85 ± 1.51^*∗*^	2.46 ± 1.58^*∗*^
	FL-TF	6.22 ± 0.86	2.96 ± 1.33^*∗*^	2.94 ± 1.70^*∗*^	2.46 ± 1.02^*∗*^

NDI	US-SNRB	24.25 ± 5.34	11.66 ± 4.87^*∗*^	13.25 ± 7.41^*∗*^	11.96 ± 3.03^*∗*^
	FL-CIESI	24.44 ± 4.78	12.68 ± 7.22^*∗*^	12.56 ± 5.63^*∗*^	12.13 ± 3.06^*∗*^
	FL-TF	24.11 ± 5.84	13.16 ± 7.12^*∗*^	12.64 ± 5.85^*∗*^	12.05 ± 4.10^*∗*^

Values are mean ± standard deviation. ^*∗*^*P* < 0.05: comparison before and after the injection. Verbal Numeric Scale (VNS), Neck Disability Index (NDI), ultrasound (US), selective nerve root block (SNRB), fluoroscopy (FL), cervical interlaminar epidural steroid injection (CIESI), transforaminal (TF).

**Table 3:** Univariable analysis for possible outcome predictors for injection effectiveness at follow-up.

Characteristic	Responders (*n* = 64)	Nonresponders (*n* = 58)	*P* value
Injection method, *n* (%)			
US-SNRB	24 (37.5)	20 (34.5)	0.942
FL-CIESI	21 (32.8)	20 (34.5)	
FL-TF	19 (29.7)	18 (31.0)	
Cause, *n* (%)			
HCD	24 (37.5)	21 (36.2)	0.882
Stenosis	40 (62.5)	37 (63.8)	
Pain duration, *n* (%)			
<6 month	24 (37.5)	15 (25.9)	0.169
>6 month	40 (62.5)	43 (74.1)	
Gender, *n* (%)			
Female	43 (67.2)	40 (69.0)	0.833
Male	21 (32.8)	18 (31.0)	
Age, *n* (%)			
≤39	8 (12.5)	6 (10.3)	0.815
40–49	14 (21.9)	14 (24.1)	
50–59	20 (31.3)	18 (31.0)	
60–69	14 (21.9)	16 (27.6)	
>70	8 (12.5)	4 (6.9)	
Number of injections, *n* (%)			
1	36 (56.3)	33 (56.9)	0.943
2	28 (43.8)	25 (43.1)	
Analgesic use, *n* (%)			
NSAID usage	32 (55.2)	38 (59.4)	0.639
Opioid usage	35 (54.7)	33 (56.9)	0.806

Ultrasound (US), selective nerve root block (SNRB), fluoroscopy (FL), cervical interlaminar epidural steroid injection (CIESI), transforaminal (TF), herniated cervical disc (HCD), non-steroidal anti-inflammatory drugs (NSAIDs).

**Table 4:** Multiple logistic regression analysis for possible outcome predictors for injection effectiveness at follow-up.

Factor	OR	95% CI	*P* value
US vs FL-guided methods	0.912	0.582–1.428	0.686
Cause (HCD, stenosis)	0.988	0.463–2.111	0.976
Sex	1.140	0.517–2.513	0.746
Age	1.006	0.972–1.041	0.729
Number of injections	1.062	0.503–2.242	0.875
Pain duration	0.863	0.725–1.028	0.099

OR: odds ratio, 95% CI: 95% confidence interval. Ultrasound (US), fluoroscopy (FL), herniated cervical disc (HCD).

## Data Availability

The datasets used and/or analysed during the current study are available from the corresponding author on reasonable request.

## Abbreviations

CESI: Cervical epidural steroid injection
SNRB: Selective nerve root block
FL: Fluoroscopy
US: Ultrasound
IL: Interlaminar
TF: Transforaminal
NDI: Neck Disability Index
VNS: Verbal Numeric Scale.

## References

[1] Radhakrishnan K., Litchy W. J., O’Fallon W. M., Kurland L. T. (1994). Epidemiology of cervical radiculopathy. *Brain*.

[2] L J., Kim Y. (2012). Outcomes of interlaminar and transforminal spinal injections. *Bulletin of the NYU Hospital for Joint Diseases*.

[3] Manchikanti L., Falco F. J. E., Diwan S., Hirsch J. A., Smith H. S. (2014). Cervical radicular pain: the role of interlaminar and transforaminal epidural injections. *Current Pain and Headache Reports*.

[4] Galiano K., Obwegeser A. A., Bodner G. (2005). Ultrasound-guided periradicular injections in the middle to lower cervical spine. *Regional Anesthesia and Pain Medicine*.

[5] Narouze S. N., Vydyanathan A., Kapural L., Sessler D. I., Mekhail N. (2009). Ultrasound-guided cervical selective nerve root block. *Regional Anesthesia and Pain Medicine*.

[6] Yamauchi M., Suzuki D., Niiya T. (2011). Ultrasound-guided cervical nerve root block: spread of solution and clinical effect. *Pain Medicine*.

[7] Jee H., Lee J. H., Kim J., Park K. D., Lee W. Y., Park Y. (2013). Ultrasound-guided selective nerve root block versus fluoroscopy-guided transforaminal block for the treatment of radicular pain in the lower cervical spine: a randomized, blinded, controlled study. *Skeletal Radiology*.

[8] Narouze S. N. (2012). Ultrasound-guided cervical spine injections. *Regional Anesthesia and Pain Medicine*.

[9] Park K. D., Lee W. Y., Nam S. H., Kim M., Park Y. (2019). Ultrasound-guided selective nerve root block versus fluoroscopy-guided interlaminar epidural block for the treatment of radicular pain in the lower cervical spine: a retrospective comparative study. *Journal of Ultrasound*.

[10] Smuck M., Maxwell M. D., Kennedy D., Rittenberg J. D., Lansberg M. G., Plastaras C. T. (2010). Utility of the anesthetic test dose to avoid catastrophic injury during cervical transforaminal epidural injections. *The Spine Journal*.

[11] Vernon H., Mior S. (1991). The neck disability index: a study of reliability and validity. *Journal of Manipulative and Physiological Therapeutics*.

[12] Hartrick C. T., Kovan J. P., Shapiro S. (2003). The numeric rating scale for clinical pain measurement: a ratio measure?. *Pain Practice*.

[13] Finlayson R. J., Etheridge J.-P. B., Tiyaprasertkul W., Nelems B., Tran D. Q. H. (2014). A prospective validation of biplanar ultrasound imaging for C5-C6 cervical medial branch blocks. *Regional Anesthesia and Pain Medicine*.

[14] Finlayson R. J., Etheridge J.-P. B., Vieira L., Gupta G., Tran D. Q. H. (2013). A randomized comparison between ultrasound-and fluoroscopy-guided third occipital nerve block. *Regional Anesthesia and Pain Medicine*.

[15] Derby R., Lee S.-H., Date E. S., Lee J.-H., Lee C.-H. (2008). Size and aggregation of corticosteroids used for epidural injections. *Pain Medicine*.

[16] Ghobadifar M. A., Akbarzadeh A., Mosallanejad Z. (2015). Which methods of epidural steroid injections is more effective in reducing the radicular pain; transforaminal or interlaminar?. *The Korean Journal of Pain*.

[17] Kim H. J., Park J. H., Shin K. M (2012). The efficacy of transforaminal epidural steroid injection by the conventional technique in far-lateral herniation of lumbar disc. *Pain Physician*.

[18] Chang Chien G. C., Candido K. D., Knezevic N. N. (2012). Digital subtraction angiography does not reliably prevent paraplegia associated with lumbar transforaminal epidural steroid injection. *Pain Physician*.

[19] Cohen S. P., Maine D. N., Shockey S. M., Kudchadkar S., Griffith S. (2008). Inadvertent disk injection during transforaminal epidural steroid injection: steps for prevention and management. *Pain Medicine*.

[20] Bilir A., Gulec S. (2006). Cauda equina syndrome after epidural steroid injection: a case report. *Journal of Manipulative and Physiological Therapeutics*.

[21] Chang-Chien G. C., Knezevic N. N., McCormick Z., Chu S. K., Trescot A. M., Candido K. D. (2014). Transforaminal versus interlaminar approaches to epidural steroid injections: a systematic review of comparative studies for lumbosacral radicular pain. *Pain Physician*.

[22] Park Y., Ahn J. K., Sohn Y. (2013). Treatment effects of ultrasound guide selective nerve root block for lower cervical radicular pain: a retrospective study of 1-year follow-up. *Annals of Rehabilitation Medicine*.

[23] Scanlon G. C., Moeller-Bertram T., Romanowsky S. M., Wallace M. S. (2007). Cervical transforaminal epidural steroid injections: more dangerous than we think?. *Spine*.

[24] Verrills P., Nowesenitz G., Barnard A. (2010). Penetration of a cervical radicular artery during a transforaminal epidural injection. *Pain Medicine*.

[25] Kaplan M. S., Cooke J., Collins J. G., Collins J. G. (2008). Intravascular uptake during fluoroscopically guided cervical interlaminar steroid injection at C6-7: a case report. *Archives of Physical Medicine and Rehabilitation*.

[26] Bose B. (2005). Quadriparesis following cervical epidural steroid injections: case report and review of the literature. *The Spine Journal*.

[27] Kranz P. G., Amrhein T. J., Gray L. (2015). Incidence of inadvertent intravascular injection during CT fluoroscopy-guided epidural steroid injections. *American Journal of Neuroradiology*.

[28] Smuck M., Fuller B. J., Chiodo A (2008). Accuracy of intermittent fluoroscopy to detect intravascular injection during transforaminal epidural injections. *Spine*.

[29] Furman M. B., Giovanniello M. T., O’Brien E. M. (2003). Incidence of intravascular penetration in transforaminal cervical epidural steroid injections. *Spine*.

[30] Manchikanti L., Malla Y., Wargo B. W., Cash K. A., Pampati V., Fellows B. (2012). A prospective evaluation of complications of 10,000 fluoroscopically directed epidural injections. *Pain Physician*.

[31] Furman M. B., Mehta A. R., Kim R. E. (2010). Injectate volumes needed to reach specific landmarks in lumbar transforaminal epidural injections. *PM&R*.

[32] Narouze S. N., Provenzano D. A. (2013). Sonographically guided cervical facet nerve and joint injections. *Journal of Ultrasound in Medicine*.

[33] Won S. J., Rhee W. I., Yoon J. S., Lee U.-Y., Ko Y. J. (2016). Ultrasound-guided lower cervical nerve root injectate volumes associated with dorsal root ganglion and epidural spread. *Journal of Ultrasound in Medicine*.

